# The Stroke That Hid in Plain Sight: Artery of Percheron Infarction With Bilateral Thalamic Involvement

**DOI:** 10.7759/cureus.117485

**Published:** 2026-10-05

**Authors:** Noor Sadiq Syed, Naveen Nagalla, Rumaisua Saeed, Sarah Robertshaw

**Affiliations:** 1 Emergency Medicine, Mid Yorkshire Teaching NHS Trust, Wakefield, GBR; 2 Internal Medicine, Doncaster and Bassetlaw Teaching Hospitals NHS Foundation Trust, Doncaster, GBR

**Keywords:** acute coma, artery of percheron infarct, bilateral paramedian thalamic infarct, bilateral thalamic infarct, posterior circulation stroke, rare cause of stroke, resus, stroke, unresponsive

## Abstract

We report the case of a 60-year-old woman with hypertension and poorly controlled insulin-treated type 2 diabetes mellitus who developed blurred vision followed by profound reduction in consciousness. She was subsequently found unresponsive at home and presented to the ED with a Glasgow Coma Scale score of 8/15, requiring emergency endotracheal intubation for airway protection. Urgent noncontrast CT of the head demonstrated no acute intracranial pathology. Following extubation, she exhibited marked drowsiness, bilateral ptosis, diplopia, and subsequently recognized vertical gaze palsy while limb strength remained relatively preserved. The ocular abnormalities initially broadened the differential diagnosis, while the persistent disturbance of consciousness prompted further investigation for central neurological causes. MRI subsequently demonstrated acute bilateral paramedian thalamic infarction with rostral midbrain involvement, consistent with an artery of Percheron (AOP) territory infarct. Following stroke team assessment, her National Institutes of Health Stroke Scale score was 3, reflecting vertical gaze palsy, left upper-limb incoordination, and dysarthria. She received secondary stroke prevention and multidisciplinary rehabilitation, with progressive improvement in consciousness, swallowing, and mobility, although residual visual symptoms persisted. The striking disparity between coma requiring airway intervention and the subsequent paucity of conventional focal neurological deficits illustrates an important diagnostic challenge of AOP infarction. A normal initial CT scan should not prematurely terminate investigation for posterior circulation ischemia when profound alteration in consciousness remains unexplained.

## Introduction

The artery of Percheron (AOP) is an anatomical variant of the posterior cerebral circulation in which a single perforating arterial trunk, typically arising from the P1 segment of one posterior cerebral artery, supplies both paramedian thalami and may additionally supply the rostral midbrain [1,2]. In the more usual anatomical configuration, the right and left paramedian thalami receive separate perforating arterial branches. Occlusion of the AOP can therefore result in bilateral paramedian thalamic infarction following obstruction of a single small vessel [1,2].

The distinction between the vascular variant and infarction within its territory is clinically important. The AOP configuration has been reported in approximately 4-12% of the population, whereas AOP territory infarctions account for approximately 0.1-2% of all ischemic strokes [1,3]. A contemporary single-center cohort identified AOP infarction in 15 of 2,830 patients with ischemic stroke, corresponding to a prevalence of 0.53% [4].

The clinical spectrum is broad and may include hypersomnolence, altered mental status, memory disturbance, vertical gaze palsy, dysarthria, ataxia, and coma [1,3,4]. These manifestations reflect the roles of the paramedian thalami and rostral midbrain in arousal, cognition, memory, and ocular motor control. Conventional lateralizing abnormalities such as unilateral limb weakness or facial asymmetry may be absent. This creates a particular diagnostic challenge in the ED. Early noncontrast CT may be unrevealing, particularly in small deep or posterior circulation infarctions, and a patient presenting primarily with impaired consciousness may initially appear to have a toxic, metabolic, infectious, epileptic, or other neurological disorder rather than acute ischemic stroke [5].

We present a patient who arrived in the ED with profound reduction in consciousness requiring emergency airway intervention despite an initially normal CT head. The distinctive feature of this case is the marked discrepancy between a life-threatening disturbance of consciousness and the relatively limited subsequent focal neurological deficit. Recognition of vertical gaze palsy ultimately provided an important localization clue, with MRI demonstrating bilateral paramedian thalamic and rostral midbrain infarction in the AOP territory.

## Case presentation

A 60-year-old woman was brought to the ED by ambulance after being found unresponsive at home. A pre-alert was received because of profound reduction in consciousness and concern regarding an acute intracranial event, and she was managed immediately in the resuscitation area. On arrival, her level of consciousness was assessed using the Glasgow Coma Scale (GCS) and was 8/15 (E1V2M5) [6]. Her airway was initially self-maintained, and bilateral air entry was present. There was no obvious external trauma, and her pupils were reactive although sluggish. Given the profound impairment in consciousness and associated risk of loss of airway protection and aspiration, the intensive care and anesthesia teams were involved early. Emergency endotracheal intubation was undertaken, and she was subsequently transferred to the ICU.

Collateral history obtained from her family established that she had complained of blurred vision during the evening before admission but had otherwise been functioning normally. On the following day, while watching television, she again experienced visual difficulty. There had been no witnessed facial asymmetry, unilateral limb weakness, or clear speech disturbance. She subsequently went to bed for what would ordinarily have been a routine afternoon nap. Several hours later, her husband was unable to wake her and contacted the emergency medical services. Her medical history included hypertension, insulin-treated type 2 diabetes mellitus, hypothyroidism, aortic stenosis, and psoriatic arthritis. Her diabetes was poorly controlled, with an inpatient glycated hemoglobin (HbA1c) concentration of 139 mmol/mol.

Urgent noncontrast CT of the head demonstrated no intracranial hemorrhage, established acute infarction, space-occupying lesion, midline shift, or herniation (Figure 1). The formal radiological conclusion was that there was no evidence of acute intracranial pathology.

**Figure 1:**
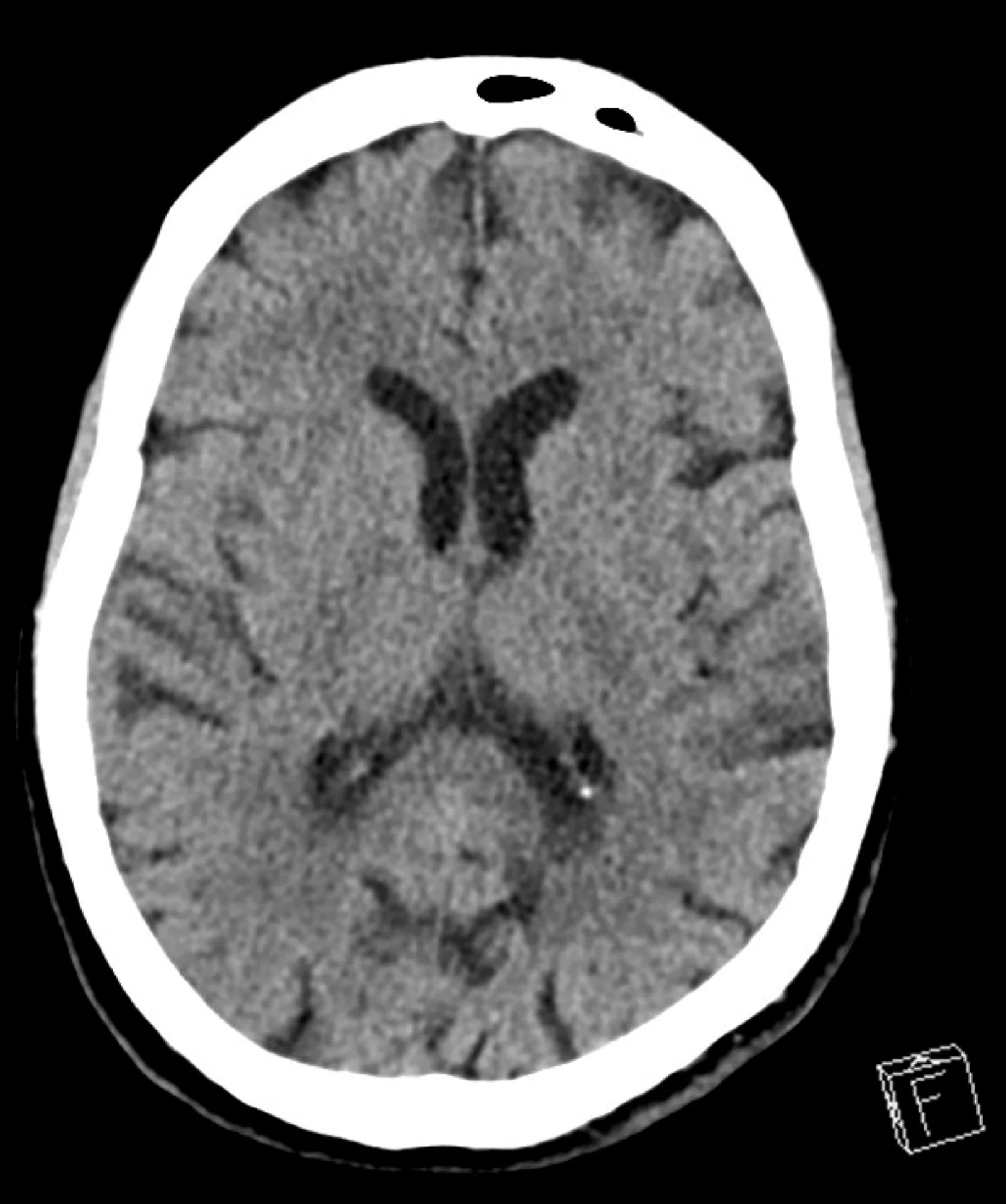
Initial noncontrast CT of the head. Noncontrast CT of the head obtained following presentation with profound reduction in consciousness. No acute intracranial hemorrhage, established infarction, space-occupying lesion, midline shift, or other acute intracranial pathology was identified.

Despite the apparently reassuring CT findings, the profound neurological impairment remained unexplained. During ICU admission, the patient remained intubated and ventilated. On the morning following admission, documented ventilatory parameters included an oxygen saturation of 97% on a fraction of inspired oxygen of 0.21, a positive end-expiratory pressure of 5 cmH₂O, and a respiratory rate of 16 breaths/min. Following reduction of sedation, she obeyed commands, demonstrated a good cough, and had preserved limb strength. She was successfully extubated later that day.

Following extubation, abnormalities of ocular function became increasingly apparent. She had marked difficulty opening both eyes, initially raising suspicion of bilateral ptosis. When able to communicate more reliably, she described diplopia and blurred vision. Serial neurological examinations demonstrated relatively preserved tone, reflexes, sensation, and limb power. The bilateral ptosis initially prompted consideration of a neuromuscular junction disorder. However, the profound disturbance of consciousness was recognized as incompatible with such a disorder as a unifying diagnosis. With persistent abnormal neurology and fluctuating alertness, central neurological causes remained the principal concern.

An infectious or inflammatory central nervous system process was also considered. Bacterial meningitis was considered less likely in the absence of significant leukocytosis or sustained fever, while viral encephalitis remained within the differential diagnosis. Empirical intravenous acyclovir was therefore commenced while neuroimaging and CSF investigations were pending. The relevant diagnostic workup included a hemoglobin concentration of 111 g/L, a white blood cell count of 8 × 10⁹/L, a neutrophil count of 6 × 10⁹/L, a platelet count of 144 × 10⁹/L, a C-reactive protein of 73 mg/L, and a procalcitonin of 0.23 ng/mL. Serum sodium was 138 mmol/L, potassium 4.4 mmol/L, urea 5.6 mmol/L, creatinine 106 µmol/L, and estimated glomerular filtration rate 49 mL/min/1.73 m². HbA1c was markedly elevated at 139 mmol/mol.

Lumbar puncture was performed on hospital day 2. CSF was clear and colorless, with no white blood cells, red blood cells, or organisms identified. CSF protein was 0.30 g/L (adult reference range: 0.15-0.45 g/L), while glucose was 5.7 mmol/L and was interpreted in relation to the concurrent plasma glucose concentration. Blood cultures demonstrated no growth. Urine culture grew Escherichia coli, and the presumed urinary tract infection was treated with oral trimethoprim. However, the treating team considered the urinary infection insufficient to account for the profound neurological presentation.

Serial neurological examination subsequently identified an important localization clue: the patient was unable to move her eyes normally in the vertical plane. The combination of pronounced hypersomnolence, bilateral ocular abnormalities, vertical gaze impairment, relatively preserved limb strength, and an initially normal CT prompted further investigation for an occult central neurological lesion.

MRI of the brain was performed on hospital day 3. Diffusion-weighted imaging demonstrated restricted diffusion within the bilateral paramedian thalami, consistent with acute infarction (Figure 2). Additional abnormality involved the rostral midbrain. The overall radiological appearances were considered diagnostic of an acute AOP territory infarction involving the bilateral thalami and rostral midbrain.

**Figure 2:**
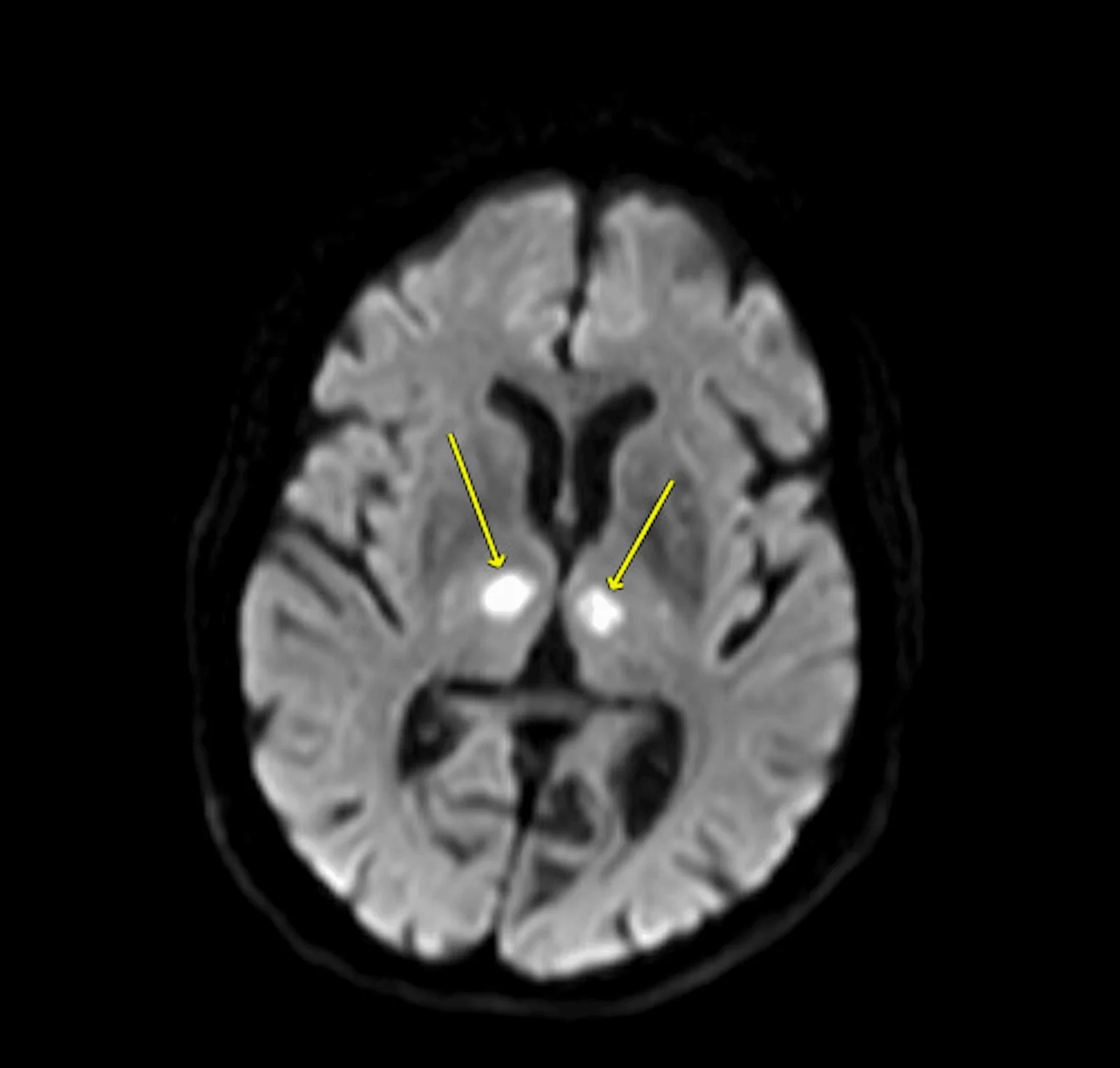
Axial diffusion-weighted magnetic resonance image demonstrating bilateral paramedian thalamic infarction. Axial diffusion-weighted magnetic resonance image demonstrating symmetrical restricted diffusion within the bilateral paramedian thalami (arrows), consistent with acute AOP territory infarction. AOP, artery of Percheron

A chronic left posterior cerebral artery territory infarct was additionally present without acute restricted diffusion and was not considered responsible for the acute presentation. Following MRI confirmation, the stroke service reviewed the patient. Her consciousness had improved to GCS 15/15. Formal neurological assessment demonstrated vertical gaze palsy, preserved power in all four limbs, preserved sensation, left upper-limb past pointing, and mild-to-moderate dysarthria. Her National Institutes of Health Stroke Scale (NIHSS) score was 3, comprising one point for gaze palsy, one point for left upper-limb ataxia, and one point for dysarthria [7].

By the time MRI established the diagnosis, several days had elapsed from the initial visual symptoms, and acute reperfusion therapy was not pursued. A CT angiogram demonstrating a proximal large-vessel occlusion was not available in the records reviewed for this report; therefore, the presence of a mechanical thrombectomy target could not be established from the available clinical information. Secondary stroke prevention was initiated with aspirin, followed by planned transition to clopidogrel, while atorvastatin was increased to high-intensity dosing.

The available stroke etiological workup did not establish a definitive mechanism. Inpatient telemetry documented sinus rhythm. Transthoracic echocardiography demonstrated preserved left ventricular systolic function with an ejection fraction greater than 55%, mild-to-moderate aortic stenosis, mild tricuspid regurgitation, and no obvious intracardiac thrombus or vegetation. A numerical inpatient low-density lipoprotein result and CT angiography of the head and neck were not available in the records reviewed. Consequently, a definitive Trial of Org 10172 in Acute Stroke Treatment (TOAST) etiological subtype was not assigned. The markedly elevated HbA1c and history of hypertension represented important vascular risk factors.

The subsequent clinical course was characterized by significant hypersomnolence, for which modafinil 100 mg twice daily was temporarily prescribed. She also developed oropharyngeal dysphagia and initially required nasogastric feeding. With multidisciplinary stroke rehabilitation and speech and language therapy, swallowing progressively improved from a modified-consistency diet to an unrestricted Level 7 diet with Level 0 thin fluids. Her alertness progressively improved, allowing modafinil to be discontinued. Mobility improved from assisted transfers to independent mobilization to the toilet. At discharge, she was alert, medically stable, eating and drinking independently, and mobilizing with substantially improved function. Residual visual disturbance persisted, including haziness and blurred vision, and neuro-ophthalmology follow-up was arranged.

## Discussion

AOP infarction presents a particular diagnostic challenge in emergency medicine because profound neurological impairment may occur without the conventional lateralizing signs that commonly trigger immediate recognition of stroke. The distinctive feature of this case was the marked disparity between an initial GCS of 8/15 requiring emergency airway intervention and the later NIHSS score of only 3, with preserved limb power and predominantly ocular, coordination, and speech abnormalities. The AOP is a single perforating arterial variant that typically arises from the P1 segment of one posterior cerebral artery and supplies both paramedian thalami, with variable additional supply to the rostral midbrain [1,2]. Although this anatomical configuration has been reported in approximately 4-12% of individuals, infarction within its territory represents only a small proportion of ischemic strokes [1,3,4].

Lazzaro et al. described four principal radiological patterns of AOP infarction. Bilateral paramedian thalamic infarction with midbrain involvement occurred in 43% of their series and represented the most frequently observed pattern [2]. The bilateral paramedian thalamic and rostral midbrain involvement demonstrated in our patient therefore corresponds to a recognized AOP distribution. The presentation is explained by the functions of the affected structures. The paramedian thalami contribute to arousal, awareness, cognition, memory, and regulation of the sleep-wake cycle. Bilateral thalamic injury can therefore result in profound hypersomnolence, stupor, or coma [1,3]. By contrast, major corticospinal pathways may remain relatively preserved, explaining why severe alteration of consciousness can coexist with little or no limb weakness. The rostral midbrain and adjacent thalamic pathways also contribute to vertical ocular movement. Vertical gaze palsy, diplopia, ptosis, and other ocular motor abnormalities are therefore recognized manifestations of AOP infarction [2-4]. In this case, the emergence of vertical gaze palsy became an important anatomical localization clue when the initial CT had failed to explain the patient’s condition.

The initial bilateral ptosis understandably broadened the neurological differential diagnosis. A neuromuscular junction disorder was considered because of the ocular findings; however, it could not account for the profound impairment of consciousness and therefore did not provide a satisfactory unifying diagnosis. Viral encephalitis was also considered during the period of unexplained altered consciousness and was treated empirically with acyclovir while further central nervous system investigations were undertaken. The initially normal CT represented a further diagnostic challenge. Noncontrast CT is essential for rapidly identifying intracranial hemorrhage and other major structural pathology but is less sensitive for small deep and posterior circulation infarctions during the early stages of ischemia. Published AOP cases have similarly demonstrated unrevealing initial CT imaging followed by diagnostic MRI [5,8].

Diffusion-weighted MRI is therefore particularly valuable when severe neurological abnormalities remain unexplained following initial CT. In our patient, restricted diffusion within the bilateral paramedian thalami established the presence of acute infarction, while rostral midbrain involvement provided an anatomical explanation for the prominent ocular motor abnormalities. Delayed or challenging recognition of AOP infarction has also been described in previous case reports and reviews, particularly when altered consciousness predominates and conventional focal neurological deficits are limited [9,10]. A recent systematic review similarly demonstrated substantial heterogeneity in clinical presentation and emphasized the diagnostic difficulty associated with this vascular syndrome [11]. Other published cases further highlight the importance of considering bilateral thalamic infarction when the presentation does not resemble a conventional hemispheric stroke [12].

The case also illustrates a limitation of conventional stroke severity assessment. Following recovery of consciousness, the patient’s NIHSS score was only 3 despite having previously required intubation because of a GCS of 8/15. This discrepancy reflects the fact that profound impairment of arousal and vertical eye movement abnormalities may produce major clinical severity without generating a correspondingly high NIHSS score. Clinical localization therefore remains essential when assessing atypical posterior circulation stroke syndromes.

The value of this case is consequently not based on rarity alone. For emergency physicians, the important diagnostic pattern is profound unexplained somnolence or coma accompanied by ocular motor abnormalities and relatively preserved limb strength. When an initial CT scan does not explain such a presentation, a bilateral thalamic or rostral midbrain process should be considered, and MRI should be pursued where clinically appropriate.

## Conclusions

AOP infarction is an uncommon but clinically important cause of bilateral paramedian thalamic stroke with variable rostral midbrain involvement. Its emergency presentation may differ substantially from conventional hemispheric stroke, with profound hypersomnolence or coma occurring despite relatively preserved limb strength and limited subsequent NIHSS abnormalities. This case demonstrates the importance of recognizing the mismatch between severe impairment of consciousness and a paucity of conventional lateralizing neurological signs. Vertical gaze disturbance, diplopia, or other ocular motor abnormalities may provide an important anatomical clue. A normal initial noncontrast CT head should not prematurely terminate neurological investigation when profound impairment of consciousness remains unexplained. In this setting, diffusion-weighted MRI can be decisive in identifying an AOP infarction or another occult posterior circulation lesion.
